# Brachial Plexus Rupture: A Neglected Trauma, Is There Any Hope?

**DOI:** 10.5334/jbsr.3415

**Published:** 2024-02-09

**Authors:** Ibtissam El Ouali, Mohamed Jiddane, Firdaous Touarsa

**Affiliations:** 1 Faculty of Medicine and Pharmacy of Rabat, Mohammed V University; Imps SOUISSI, Rabat 10100. Morocco; 2 Faculty of Medicine and Pharmacy of Rabat, Mohammed V University; Imps SOUISSI, Rabat 10100. Morocco; 3 Faculty of Medicine and Pharmacy of Rabat, Mohammed V University; Imps SOUISSI, Rabat 10100. Morocco

**Keywords:** Brachial plexus, trauma, MRI

## Abstract

*Teaching point:* Magnetic resonance imaging (MRI) has significantly improved the evaluation of brachial plexus injuries, offering new possibilities for microsurgical repair and contributing to the functional prognosis.

## Case Report

**Figure 1:**
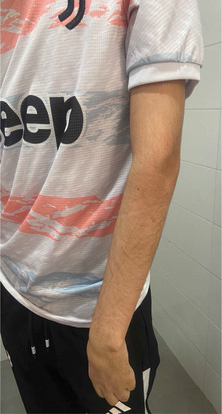
A 26-year-old male presenting with neglected weakness of the left upper limb associated with dysesthesia and advanced atrophy following a motor vehicle accident 3 years ago.

We report the case of a 26-year-old male presenting with neglected weakness of the left upper limb associated with dysesthesia and advanced atrophy following a motor vehicle accident 3 years ago (Figure 1).

**Figure 2:**
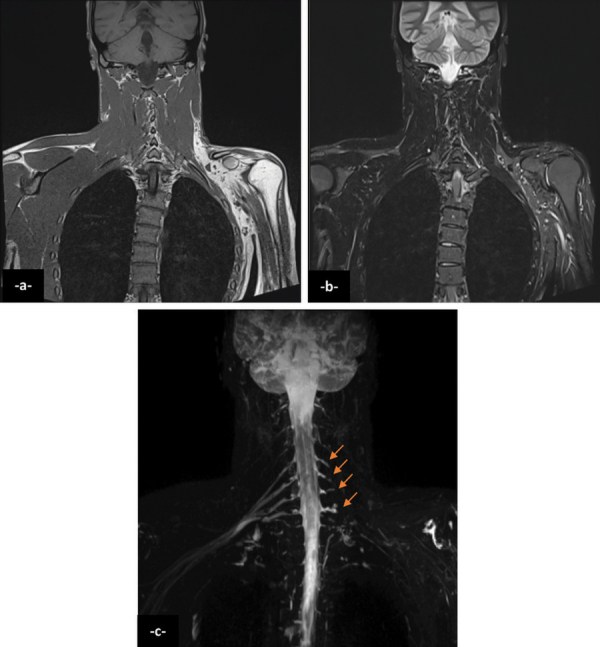
Coronal T1 and T2 FAT SAT magnetic resonance imaging (MRI) of the brachial plexus were performed, showing fatty infiltration of the left shoulder and upper limb muscles (a,b). Contrast-enhanced coronal 3D SPACE STIR showed complete discontinuity of the left brachial plexus from C4 to C7 at the level of post-ganglionic segments with retraction of distal branches (c, arrows).

Coronal T1 and T2 FAT SAT magnetic resonance imaging (MRI) of the brachial plexus were performed, showing fatty infiltration of the left shoulder and upper limb muscles (Figure 2a and 2b).

Contrast-enhanced coronal 3D SPACE STIR showed complete discontinuity of the left brachial plexus from C4 to C7 at the level of post-ganglionic segments with retraction of distal branches (Figure 2C, arrows). Surgical grafting was performed, however, with an incomplete functional recovery.

## Comments

The brachial plexus originates in the posterior cervical triangle by the union of the ventral rami of the 5th, 6th, 7th, and 8th cervical nerve roots and the 1st thoracic nerve root, thus innervating the upper extremity.

A global increase in the incidence of traumatic brachial plexus injuries has been reported with a significant predominance in males. Motorcycle accidents with major traction injuries are the cause of 70% of traumatic cases [1].

The level of the injury determines the surgical prognosis. Lesions proximal to the dorsal root ganglion and preganglionic avulsion decrease the likelihood of nerve grafting success. Post-ganglionic injuries have a somewhat better prognosis.

Identification of pre-ganglionic lesions requires a dedicated MRI, preferably at 3T.

Coronal T2 FS imaging contributes to the assessment of edema and nerve contusions, and axial T2WI enable to evaluate the integrity of the plexus from the root ganglion to the distal ends in order to assess nerve root avulsion and pseudomeningocele. Stretching (incomplete avulsion) presents as differential enhancement of the rootlets.

Post-ganglionic injuries occur proximally at the scalene triangle and may involve the trunks or distal branches in a variable way. Distal nerve retraction is observed in complete ruptures.

In the acute setting, CT visualizes skeletal lesions and hematomas, and CT-angiography is indicated when vascular lesions are suspected.

Pre-ganglionic injuries may require early (<3 months) reconstruction, whereas postganglionic injuries may be repaired after a longer period of observation.

Current surgical options include nerve transfers (neurotization, nerve grafting, and neurolysis), tendon or muscle transfers with a better functional result for post-ganglionic injuries even after 9 months of the traumatic event.

## Conclusion

Brachial plexus injuries have become increasingly frequent in the young population, and dedicated MRI protocols have contributed to improve the prognosis for functional recovery by early and accurate assessment of nerve lesions. Accurate interpretation and detailed reporting of the nerve injuries are crucial for therapeutic management.
